# Migraine and Its Equivalents: What Do They Share? A Narrative Review on Common Pathophysiological Patterns

**DOI:** 10.3390/life11121392

**Published:** 2021-12-12

**Authors:** Ilaria Frattale, Claudia Ruscitto, Laura Papetti, Fabiana Ursitti, Giorgia Sforza, Romina Moavero, Michela Ada Noris Ferilli, Samuela Tarantino, Martina Balestri, Federico Vigevano, Luigi Mazzone, Massimiliano Valeriani

**Affiliations:** 1 Child Neurology and Psychiatry Unit, Systems Medicine Department, Tor Vergata University, Hospital of Rome, 00165 Rome, Italy; ilariafrattale@libero.it (I.F.); claudia.ruscitto@opbg.net (C.R.); romina.moavero@opbg.net (R.M.); luigi.mazzone@ptvonline.it (L.M.); 2 Neurology Unit, Department of Neuroscience, Bambino Gesù Children Hospital, IRCCS, 00165 Rome, Italy; laura.papetti@opbg.net (L.P.); fabiana.ursitti@opbg.net (F.U.); giorgia.sforza@opbg.net (G.S.); michela.ferilli@opbg.net (M.A.N.F.); samuela.tarantino@opbg.net (S.T.); martina.balestri@opbg.net (M.B.); federico.vigevano@opbg.net (F.V.); 3 Center for Sensory-Motor Interaction, Aalborg University, 9220 Aalborg Øst, Denmark

**Keywords:** migraine, migraine equivalents, pathophysiology, migraine syndrome of childhood

## Abstract

Migraine is the first in order of frequency of the neurological disorders, affecting both adult and paediatric populations. It is also the first cause of primary headaches in children. Migraine equivalents are periodic disorders that can be associated with migraine or considered as prognostic features of a future migraine manifestation. Despite the mechanisms underlying migraine and its equivalents are not entirely clear, several elements support the hypothesis of common pathophysiological patterns shared by these conditions. The aim of this review is thus to analyze the literature in order to highlight which currently known mechanisms may be common between migraine and its equivalents.

## 1. Introduction

Headache is an extremely common disorder that might affect people at any age. Regarding pediatric age, as also established by the World Health Organization (WHO), migraine is the third most disabling condition [1] with a prevalence in childhood and adolescence that ranges from 7.7% to 17.8% and a gender difference of 3.7% (9.7% in females vs 6.0% in males) [2,3,4]. Migraine is a neurological condition that involves multiple brain areas responsible for controlling sensory, affective, cognitive and autonomic functions. Migraine attacks are characterized by a variety of symptoms involving the sensory system with symptoms such as phonophobia, osmophobia, photophobia, allodynia and muscle pain; autonomic systems with nausea, vomiting, ptosis, lacrimation, yawning, nasal congestion or changes in urination and defecation; cognitive impairment with transient amnesia, attention deficit and word-finding difficulties; affective areas with irritability and depression [5].

Episodic syndromes that can be associated with migraine, also known as migraine equivalents, are a set of periodic or paroxysmal manifestations that can be associated with migraine or be considered as prognostic factors of a future migraine manifestation [6]. Children with episodic syndromes show normal neurological examination, family history of migraine (65–100%) and future development of migraine in a percentage of cases ranging from 25 to 70% [7,8]. Several elements support the hypothesis that migraine and episodic syndromes associated with migraine share common pathophysiological patterns. In fact, both conditions have periodic and paroxysmal presentation [9,10]. Associated symptoms such as phonophobia, photophobia, vomiting, nausea and pallor could subsist in both episodic syndromes and migraine attacks [9,11,12,13,14]. Furthermore, a familial and genetic background is typical of both migraine and episodic syndromes [12,15,16,17,18,19,20]. Triggers can be psychological and physical, and often common to both conditions [12,15,16,17,18,21] as the same typical neurophysiological alterations [22,23]. Lastly, migraine preventive treatment can be effective also in migraine equivalents [12,14,24]. Episodic syndromes mentioned in the third edition of the International Classification of Headache Disorders (ICHD3) [11] include recurrent gastrointestinal disturbance, cyclical vomiting syndrome, abdominal migraine, infantile colic, benign paroxysmal torticollis and benign paroxysmal vertigo (Table 1). Other conditions probably associated with migraine are motion sickness, periodic sleep disorders (such as sleep walking, sleep talking, night terrors and bruxism) and leg pain [11].

This review aims to underline the pathophysiological mechanisms common to migraine and its equivalents.

## 2. Pathophysiology

### 2.1. Recurrent Gastrointestinal Disturbance

Recurrent gastrointestinal disturbance includes abdominal migraine and cyclical vomiting syndrome [11]. It consists of multiple (≥5) attacks of nausea, vomiting and abdominal pain, occurring consistently over time or at predictable intervals, that may be associated with migraine.

Abdominal migraine is typical of schoolchildren [25], affecting children from the age of 7 with a peak around 10 years of age [26] and a prevalence of 4–15% [27]. It consists of attacks of abdominal pain with an intensity that could vary, lasting from 1 to 72 h, with a complete resolution of symptoms between them [28]. Abdominal pain is typically poorly localized or in the periumbilical or midline area, with concomitant vomiting, nausea, anorexia or paleness [11]. Vomiting is not as important as in cyclic vomiting [29] and headaches are characteristically absent. It may happen that the episode of abdominal migraine is preceded by visual symptoms, sensory symptoms, language impairment and muscle weakness [30]. Both abdominal migraine and migraine present similar manifestations during attacks, including vomiting, nausea, anorexia, asthenia, limb pain and paleness and they share the same trigger factors, such as psychological and physical stress [31]. The diagnosis is one of exclusion, after having carried out other investigations and after collecting an accurate anamnesis [11].

The pathophysiological mechanisms underlying abdominal migraine are not yet fully understood [32]. A common pattern has been shown between genetic alterations of mitochondrial diseases and hypothalamic–pituitary alterations [33] (Table 2). Both the digestive and nervous systems are known to share the same embryological origin and thus an influence on each other [6]. Some authors consider it as a variant of cyclic vomiting [17] while other authors consider CVS and abdominal migraine the same condition that manifests itself differently, while others relate it to abdominal epilepsy, mostly in association with abnormal EEG findings [17]. Familiarity of migraine is present in 79% of patients [34]. In a 10-year follow-up longitudinal study of 54 children with abdominal migraine, symptoms resolution was found in 69% of the patients and their persistence until late adolescence in 31%, while 46% of patients developed typical migraine headaches [34].

CVS affects about 2% of school-age children [15], with an age of onset from 4 to 7 years [35]. CVS is characterized by cyclic stereotyped pattern of episodes of vomiting and severe nausea accompanied by pallor and lethargy with resolution between attacks [6]. The episodes typically consist of four phases: a phase of well-being between one crisis and another, the “premonitory phase,” the “emetic phase,” and finally the “recovery phase.” The duration of these phases can be variable, ranging from hours to days. Some patients may not report all stages, without showing the premonitory or recovery phase [36]. Vomiting usually resolves from 40 to 60% of patients from 10 years of age [6], but the persistence of symptoms is also largely described in adulthood [25]. CVS presentation before the age of 6.7 is considered a predictor of future adult migraine manifestation [6]. Subsequent migraine is diagnosed in a percentage of patients ranging from 40% [6,34] to 79% [37].

The pathophysiology of CVS is not fully known (Table 2), but it is probably multifactorial, involving abnormalities of the emetic reflex [38]. The currently proposed models include different mechanisms, such as alteration of autonomic system [39], metabolism disorders including mitochondrial and fatty acid suggested by the high rates of maternal genetic transmission [40,41,42], gastric dysmotility [43,44,45,46,47], an altered release at the hypothalamic–pituitary level of factors, such as corticotropin and release of vasopressin [33], endocannabinoid system dysfunction [38,48,49] and neuronal hyperexcitability [38]. Migraine and CVS share co-involvement of calcitonin gene-related peptide (CGRP) and serotonin in the modulation of cortical spreading depression, cortical pain transmission and intestinal microbiota [50], hyperactivation of the parasympathetic and sympathetic nervous systems and alterations of adrenergic autonomic system, both in children and adults [51]. Vomiting involves area postrema in the central nervous system and peripheral nervous system such as vagal and non-vagal pathways, and the gastrointestinal tract. Signals are mediated by neurotransmitters, mainly 5-hydroxytryptamine and substance P (SP) [51].

### 2.2. Infant Colic

Infant colic affects 5–19% of infants [52,53], with a prevalence culminating approximately at 6 weeks after birth and a resolution around 3 months of age [13]. These episodes are characterized by an excessive crying of healthy infants towards evening, suggesting a temporal pattern [29], for at least 3 h per day 3 days per week in the preceding 3 weeks [54]. Eighty-six percent of patients with infant colic have a family history of migraine (first-degree relative) [55]. The etiology is not completely known. Different causes have been hypothesized: (1) crying is the response to painful gut contractions caused by allergy to cow’s milk, lactose intolerance, or excess gas [53]; (2) colic is a behavioral problem [56,57]; (3) excessive crying is caused by headaches or represents an abdominal migraine variant [55] (Table 2). As a demonstration of the latter theory, several studies observed that a percentage ranging from 52 to 66% of patients with migraine had a medical history of infantile colic, compared to 20–23% of healthy controls [13,55,58]. The genetic predisposition to migraine could explain the extreme sensitiveness of these infants to different stimuli. Crying could be the expression of this hypersensitivity influenced by circadian biology. This is could be explained by the fact that colic occurs mostly in the evening [6] and resolves around 3 months of age, when a rhythmic excretion of endogenous melatonin develops in the infant’s brain [53,54,59]. In a retrospective study conducted by Jan et al. [55], 52% of children with migraine headaches during childhood had previously suffered from infantile colic. Gelfand et al. [54] reviewed five studies and 10 publications from Europe, Saudi Arabia and United States and 66% of children with migraine were found to have a medical history of infant colic.

### 2.3. Benign Paroxysmal Vertigo

Benign Paroxysmal vertigo is considered by some authors as an early form of migraine with brainstem aura (basilar migraine) [60]. Age of onset is between 2 and 5 years of age and the prevalence is independent of gender. The episodes last from few seconds to minutes with a rapid onset of the symptoms. Children in good health conditions develop disabling vertigo suddenly, with no triggers or prodromal symptoms. Some appear sweaty and pale, and vomiting or nystagmus may also be associated. Vertigos may appear in any position. Patients do not show headache or loss of consciousness and within several minutes, the symptoms disappear leaving no deficit [28]. Interestingly, benign paroxysmal vertigo constitutes an age-specific manifestation of defective neuronal calcium channel activity (Table 2). Criteria for diagnosis of benign paroxysmal vertigo include normal EEG pattern and assessment of audiometric and vestibular functions [11]. A percentage ranging from 39 to 100% reported a family history of migraine [16,60,61,62,63].

### 2.4. Benign Paroxysmal Torticollis

Benign paroxysmal torticollis is a benign condition, with complete and spontaneous remission, characterized by recurrent episodes of usually alternating side head tilt [11]. The age of onset is during the first year of life, typically in the first 6 months, with episodes recurring every 45–75 days with a certain periodicity, lasting 4.5–6 days, improving by age 2 years, and resolving by age 3 or 4. In 12% of cases, migraine was reported [8]. The etiology of this disorder is unknown, although is considered by some authors as a variety of migraine with brainstem aura [28]. In this condition, an association with a mutation of the CACNA1A gene is common. This specific gene is responsible for encoding the main subunit of the alpha-1 pore which is the main voltage-dependent calcium channel [64], also involved in familial hemiplegic migraine type 1 [65] (Table 2). The same mutation was also found in the subjects with absence or episodic ataxia [66,67]. Other mutations involved in familial hemiplegic migraines are: ATP1A, encoding a sodium–potassium-transporting ATPase subunit in FHM type 2 [68,69]; SCN1A, a voltage-gated sodium channel subunit, in FMH tipe 3 [70] while no mutation has been identified to date in FMH type 4. A PRRT2 mutation was described in a family with benign paroxysmal torticollis, hemiplegic migraine and paroxysmal kinesigenic dystonia [71]. This suggests that some cases of benign paroxysmal torticollis may be due to a channelopathy [29]. In a longitudinal study on patients with history of benign paroxysmal torticollis, we showed that migraine was diagnosed in 81% of the cases, with a mean age at onset of 5 years [37].

### 2.5. Other Symptoms Related to Migraine

Among the migraine variants, motion sickness, otherwise known as travel sickness, is also a frequent feature. This is a result of mismatching between vestibular system and visually perceived movement [72]. It is characterized by the same symptoms that usually occur during migraine attacks such as dizziness, nausea, sometimes vomiting, fatigue and pallor [73,74]. Some authors believe that motion sickness could be linked to a low serotonin level in the brain [75]. Motion sickness share common pathophysiological mechanisms with migraine (Table 2), presenting in about half of people with migraines [76]. Firstly, vestibular instability, possibly due to an impaired calcium ion channel mainly expressed in the inner ear and in the brain, could lead to reversible hair cell depolarization, resulting in otoneurological symptoms experienced both in migraine and in motion sickness [77]. Secondly, in both motion sickness and migraine attack, gastrointestinal hypersensitivity and nausea arise from the activation of a pathway involving the dorsal nucleus of the vagus and the nucleus tractus solitarius, called as vomiting center, connected with vestibular nuclei. In the migraine attack, nausea and vomiting may depend on the close functional interconnection between the trigemino-vascular system and the nucleus tractus solitarius [77]. In case of motion sickness, vestibular nuclei can activate the nucleus tractus solitarius through the cerebellum [78]. Barabas et al. reported that one half of pediatric migraine patients had motion sickness compared with 7% of non-migraine pediatric patients [79]. In a study conducted by Jan on 29 children with recurrent vomiting, one half had motion sickness and showed a higher likelihood to develop migraines, as compared to control subjects. In fact, 73% of children with motion sickness developed migraines, compared to 35% of children without motion sickness [80]. In a retrospective study, we investigated the prevalence of migraine equivalents in 830 migraine pediatric patients. Among them, 70.3% had migraine equivalents, 30% showed more than one equivalent, and 40.5% suffered from motion sickness [24]. In particular, motion sickness was the third more prevalent migraine equivalent, after abdominal migraine and recurrent limb pains [24].

Recurrent limb pains are recurrent short episodes of pain lasting until 72 h leading to an interruption of activities. Pain is localized deeply in the extremities of arms or legs. Limb pain is self-limiting and is also defined as “growing pain”. Pain benefits from resting, analgesic administration or arms rubbing [12]. Secondary causes of limb pain such as sport injuries, physical exercise or orthopedic causes should be investigated [81]. One third of cases presents associated headache and abdominal pain [12]. A study enrolling 2165 school-age children 5–15 years old showed that 2.6% suffered from limb pain with monthly recurrent episodes lasting an average of 10 h. Associated symptoms were nausea and anorexia. In first degree relatives, a clinical history of migraine was more common than in the matched control group [18]. We observed that 44% of our 830 migraine patients referred to limb pain [24]. Several authors underlined the strict temporal relationship between recurrent limb pain and migraine [24,82,83,84,85,86,87]. Drugs used for migraine prophylaxis are also effective in limb pain [88].

## 3. Discussion

Close connection between migraine and its equivalents has been based on putatively common pathogenetic mechanisms and high prevalence of migraine in subjects who presented equivalents. Indeed, the simultaneous presence of more equivalents at different ages in the same subject who will then develop migraine suggests an age-dependent evolution of symptoms before the onset of migrainous headache [24,37]. Although equivalents have been considered specific to children and adolescence with migraine [58,89], some studies suggest that these symptoms can also be found in the personal history of subjects with subsequent tension type headache [24,37]. The last observation supports that, regarding pediatric age, the hypothesis of a strict division between migraine and tension-type headache is not probably appropriate, considering that both conditions can be seen as two phenotypes of the migrainous syndrome. Several studies found evidence that in children, migraine and tension-type headache appear to be different features of the same spectrum of benign headache [24].

### 3.1. How Do We Know That Episodic Syndromes Are Associated with Migraine?

The gastroenterological episodic syndromes (abdominal migraine, CVS and infant colic) may share a common pathogenetic mechanism with migraine, due to the same embryologic origin of both enteric and central nervous tissues, which can exert direct effects on each other [90]. In the trigger mechanisms of migraine, a crucial role is played by CGRP (calcitonin gene-related peptide) and PACAP (pituitary adenylate cyclase-activating peptide), that mediate vasodilation, and serotonin, that mediates sensitization of the trigeminal neurons. During a migraine attack, activation of the trigeminovascular system leads to the release of CGRP from the trigeminal endings. CGRP determines vasodilation of intracranial arteries, modulates neuronal excitability by facilitating pain transmission, and activates neurogenic inflammation [91]. The CGRP neuropeptide shows two isoforms, α and β. The α isoform is involved in the pathophysiology of migraine pain, while the β isoform is implied in the motility of the enteric system from which is expressed, through the encoding of a different gene [92]. It also appears that CGRP induces neurogenic inflammation of sensory neurons in the gastrointestinal system [93]. Moreover, PACAP can be involved in the pathophysiology of the functional gastrointestinal disorders, since its lack in modified mice reduces the intensity of visceral pain [94]. Serotonin could be even more important in mediating gastrointestinal symptoms, since it is implied in smooth muscle motility processes and visceral sensations [95]. The association between gastrointestinal symptoms and migraine is supported by genetic studies. In their metanalysis, Gormley et al. [96] identified 44 independent single-nucleotide polymorphisms (SNPs), significantly associated with migraine risk. These SNPs map to 38 distinct genomic loci enriched for genes expressed in vascular and smooth muscle tissues, especially in the gastrointestinal system.

Unfortunately, the pathophysiological mechanisms of other migraine equivalents, such as leg pain, are not clearly known. In these cases, the association between these symptoms and migraine can be only argued on the base of two elements: (1) the clinical observation of the presence of these symptoms in the personal clinical history of patients with migraine and (2) the development of migrainous headache in children initially referred for equivalents.

One of the latest theories that clarifies some aspects of migraine concerns central and peripheral sensitization. Sensitization is a prolonged activation of peripheral and central nociceptors, which can explain both the associated symptoms and why a migraine attack can last for a long time and lead to chronic migraine [97]. This theory is based on the concept that migraine is triggered by a stimulation of the peripheral dural blood vessels with activation of the trigeminal system through the first, second and third order trigeminal neurons and structures, such as the brainstem and the diencephalon [98]. This cascade of events is believed to be responsible for both the throbbing quality of pain and associated symptoms, such as sleep disturbances, nausea, vomiting and cognitive symptoms [99]. These disturbances are common also to migraine equivalents, thus they could be due to the same pathophysiological mechanisms initiated by sensitization phenomena.

### 3.2. Do Shared Pathophysiological Mechanism Lead to Common Treatments?

There is no specific therapy for migraine equivalents. The same preventive and attack drugs can be used because of the pathophysiological background they share with migraines.

In cyclic vomiting syndrome, rehydration is important. In the acute phase it is possible to use ondansetron [17]. There is some evidence on the use of amitriptyline, cyproheptadine and propranolol [100,101] as prophylaxis. In abdominal migraine, it is possible to use triptans during the attack [102] and flunarizine is a good therapeutic option if prophylaxis is needed [103].

### 3.3. Future Perspectives

The investigation on the episodic syndromes that may be associated with migraine remains to be concluded. Here, we suggest some possible ways in which our confidence in a close relationship between equivalents and migraines could be made more reliable.

Firstly, while most studies calculated the prevalence of episodic syndromes in children with headache, longitudinal investigations on children with migraine equivalents, in order to predict a possible future migraine and/or tension-type headache, were rarely performed [13,37]. More longitudinal and possibly multicentric studies are needed to support the association between episodic syndromes and a consequent development of primary headaches and unveil the pathophysiological mechanisms shared by these conditions.

Secondly, some neurophysiological aspects, such as habituation deficiency, should be investigated in children with equivalents before the onset of migraine. Electrophysiological studies have shown that an altered cerebral cortex excitability and abnormal central information processing may be common in both adults and children with migraine [104,105]. In particular, a reduced evoked potential habituation has been demonstrated in children with migraine also using different evoked potential modalities [104,106,107,108,109,110]. The demonstration of a reduced habituation of the evoked potential amplitude also in children with episodic syndromes would represent a solid linkage between these conditions and migraine.

In a recent case control study conducted in Greece on a pediatric population [111], a possible protective role of the Val66Met polymorphism in the pathogenesis of migraine was investigated. It would act by reducing the release of BDNF (brain-derived neurotrophic factor) [105] and participating in the regulation of pain signalling and central sensitization [112,113]. BDNF is co-expressed at the level of trigeminal ganglion neurons with CGRP [114]. The authors concluded that the presence of polymorphism is associated with a reduced risk of migraine. If Val66Met polymorphism and other genetic substrates, associated with migraine development, could be related also to the episodic syndromes; this would shed light on the pathophysiology of the last ones.

Lastly, structural changes have been demonstrated in the brains of children and adolescent with migraines [115]. In this MRI study, we showed that, as compared with control subjects, young patients with migraine have a decreased gyrification index in the left superior and inferior parietal lobules, implied in the nociceptive pathway, in the supramarginal gyrus, involved in the cognitive evaluation of pain, in the right postcentral gyrus, in the right superior, middle and transverse temporal gyri and in the right supramarginal gyrus. Cortical thickness was found to decrease in patients older than 12 years, compared to the younger patients. Lastly, compared to healthy controls, an increased cortical thickness in the pars opercularis of the inferior frontal gyrus, was found in patients with migraine attacks characterized by nausea and/or vomiting. In particular, a congenital predisposition to develop migraine appears to be associated with differences in cortical gyrification. It would be interesting to have neuroimaging studies available before the onset of headache in children with migraine equivalents in order to assess the possible existence of patterns similar to those demonstrated in children with migraines.

## 4. Conclusions

In spite of a largely incomplete knowledge of the shared pathophysiology, evidence that episodic syndromes are associated with migraines is considered high enough that some authors, including ourselves, have proposed the use the term “migraine syndrome of childhood” to include both migraine equivalents and migrainous headache [24,116]. However, some issues are still far to be clear and should be object of future research. We do not know why migraine equivalents develop preferentially in pediatric age, although they can sometimes continue into adulthood. Moreover, it is unclear why not all patients with migraines and/or tension-type headaches have migraine equivalents in their personal clinical history.

Episodic syndromes could possibly contribute to define different migraine phenotypes, which could be considered when testing the efficacy of treatments. This can hopefully allow us to understand why a significant number of patients is unresponsive to a certain treatment and to tailor the therapy according to the individual migrainous phenotype.

## Figures and Tables

**Table 1:** Episodic syndromes that may be associated with migraine (ICHD3 criteria).

Recurrent gastrointestinal disturbance	A. At least five attacks with distinct episodes of abdominal pain and/or discomfort and/or nausea and/or vomiting B. Normal gastrointestinal examination and evaluation C. Not attributed to another disorder.
Cyclic vomiting syndrome	A. At least five attacks of intense nausea and vomiting, fulfilling criteria B and C B. Stereotypical in the individual patient and recurring with predictable periodicity C. All of the following: nausea and vomiting occur at least four times per hour/attacks last for 1 h, up to 10 days/attacks occur 1 week apart D. Complete freedom from symptoms between attacks E. Not attributed to another disorder
Abdominal migraine	A. At least five attacks of abdominal pain, fulfilling criteria B–D B. Pain has at least two of the following three characteristics: midline location, periumbilical or poorly localized/dull or ‘just sore’ quality/moderate or severe intensity C. At least two of the following four associated symptoms or signs: anorexia/nausea/vomiting/pallor D. Attacks last 2–72 h when untreated or unsuccessfully treated E. Complete freedom from symptoms between attacks F. Not attributed to another disorder
Infantile colic	A. Recurrent episodes of irritability, fussing or crying from birth to four months of age, fulfilling criterion B B. Both of the following: episodes last for 3 h/day/episodes occur on 3 days/week for 3 weeks C. Not attributed to another disorder
Benign paroxysmal vertigo	A. At least five attacks fulfilling criteria B and C B. Vertigo1 occurring without warning, maximal at onset and resolving spontaneously after minutes to hours without loss of consciousness C. At least one of the following five associated symptoms or signs: nystagmus/ataxia/vomiting/pallor/fearfulness D. Normal neurological examination and audiometric and vestibular functions between attacks E. Not attributed to another disorder
Benign paroxysmal torticollis	A. Recurrent attacks1 in a young child, fulfilling criteria B and C B. Tilt of the head to either side, with or without slight rotation, remitting spontaneously after minutes to days C. At least one of the following five associated symptoms or signs: pallor/irritability/malaise/vomiting/ataxia D. Normal neurological examination between attacks E. Not attributed to another disorder

**Table 2:** Common pathophysiological mechanisms with migraine.

Cyclic vomiting syndrome	CGRP and serotonin involved in the modulation of cortical spreading depression, cortical pain transmission and intestinal microbiota [50] hyperactivation of the parasympathetic and sympathetic nervous systems and alterations of adrenergic autonomic system [51]
Abdominal migraine	mitochondrial disease gene mutations and hypothalamic-pituitary-axis dysfunction [33]
Infantile colic	hypersensitivity influenced by circadian biology and CGRP modulates the sensory activity that, on its turn, is potentially involved in the pathogenesis of abdominal pain by inducing the neurogenic inflammation of sensory neurons in the gut [50]
Benign paroxysmal vertigo	defective neuronal channel activity [28]
Benign paroxysmal torticollis	mutation of calcium ion, sodium/potassium pump and sodium transporter (CACNA1A, ATP1A2 and SCN1A) [66,67,68,69,70]
Motion sickness	vestibular instability due to a defective calcium ion channel, involvement of vomiting center [77]

## Data Availability

Not applicable.

## References

[1] GBD (2018). 2017 Disease and Injury Incidence and Prevalence Collaborators. Global, regional, and national incidence, prevalence, and years lived with disability for 354 diseases and injuries for 195 countries and territories, 1990–2017: A systematic analysis for the Global Burden of Disease Study 2017. Lancet.

[2] Abu-Arafeh I., Razak S., Sivaraman B., Graham C. (2010). Prevalence of headache and migraine in children and adolescents: A systematic review of population-based studies. Dev. Med. Child Neurol..

[3] Kienbacher C., Wöber C., Zesch H., Hafferl-Gattermayer A., Posch M., Karwautz A., Zormann A., Berger G., Zebenholzer K., Konrad A. (2006). Clinical Features, Classification and Prognosis of Migraine and Tension-Type Headache in Children and Adolescents: A Long-Term Follow-Up Study. Cephalalgia.

[4] Özge A., Bugdayci R., Sasmaz T., Kaleagasi H., Kurt Ö., Karakelle A., Tezcan H., Siva A. (2002). The Sensitivity and Specificity of the Case Definition Criteria in Mersin. Cephalalgia.

[5] Burstein R., Noseda R., Borsook D. (2015). Migraine: Multiple Processes, Complex Pathophysiology. J. Neurosci..

[6] Lagman-Bartolome A.M., Lay C. (2015). Pediatric Migraine Variants: A Review of Epidemiology, Diagnosis, Treatment, and Outcome. Curr. Neurol. Neurosci. Rep..

[7] Teixeira K.C.S., Montenegro M.A., Guerreiro M.M. (2013). Migraine Equivalents in Childhood. J. Child Neurol..

[8] Rosman N.P., Douglass L.M., Sharif U.M., Paolini J. (2009). The Neurology of Benign Paroxysmal Torticollis of Infancy: Report of 10 New Cases and Review of the Literature. J. Child Neurol..

[9] Winner P. (2013). Migraine-Related Symptoms in Childhood. Curr. Pain Headache Rep..

[10] Al-Twaijri W.A., Shevell M.I. (2002). Pediatric migraine equivalents. Pediatr. Neurol..

[11] Olesen J. (2018). Headache Classification Committee of the International Headache Society (IHS) The International Classification of Headache Disorders, 3rd edition. Cephalalgia.

[12] Cuvellier J.-C., Lépine A. (2010). Childhood Periodic Syndromes. Pediatr. Neurol..

[13] Gelfand A.A. (2013). Migraine and childhood periodic syndromes in children and adolescents. Curr. Opin. Neurol..

[14] Forbes D., Fairbrother S. (2008). Cyclic nausea and vomiting in childhood. Aust. Fam. Physician.

[15] Abu-Arafeh I., Russell G. (1995). Cyclical Vomiting Syndrome in Children: A Population-Based Study. J. Pediatr. Gastroenterol. Nutr..

[16] Abu-Arafeh I., Russell G. (1995). Paroxysmal Vertigo as a Migraine Equivalent in Children: A Population-Based Study. Cephalalgia.

[17] Li B.U., Lefevre F., Chelimsky G.G., Boles R.G., Nelson S.P., Lewis D.W., Linder S.L., Issenman R.M., Rudolph C.D. (2008). North American Society for Pediatric Gastroenterology, Hepatology, and Nutrition Consensus Statement on the Diagnosis and Management of Cyclic Vomiting Syndrome. J. Pediatr. Gastroenterol. Nutr..

[18] Abu-Arafeh I., Russell G. (1996). Recurrent limb pain in schoolchildren. Arch. Dis. Child..

[19] Uneri A. (2004). Migraine and Benign Paroxysmal Positional Vertigo: An Outcome Study of 476 Patients. Ear Nose Throat J..

[20] Hernandez-Latorre A.M., Roig M. (2000). Natural History of Migraine in Childhood. Cephalalgia.

[21] Prakash S., Shah N.D., Dholakia S.Y. (2009). Recurrent Limb Pain and Migraine: Case Reports and A Clinical Review. Cephalalgia.

[22] Jernigan S.A., Ware L.M. (2008). Reversible Quantitative EEG Changes in a Case of Cyclic Vomiting: Evidence for Migraine Equivalent. Dev. Med. Child Neurol..

[23] Mortimer M.J., Good P.A. (1990). The VER as a Diagnostic Marker for Childhood Abdominal Migraine. Headache J. Head Face Pain.

[24] Tarantino S., Capuano A., Torriero R., Citti M., Vollono C., Gentile S., Vigevano F., Valeriani M. (2014). Migraine Equivalents as Part of Migraine Syndrome in Childhood. Pediatr. Neurol..

[25] Lewis D.W. (2009). Pediatric Migraine. Neurol. Clin..

[26] Russell G., Abu-Arafeh I., Symon D.N.K. (2002). Abdominal migraine: Evidence for existence and treatment options. Pediatr. Drugs.

[27] Carson L., Lewis D., Tsou M., McGuire E., Surran B., Miller C., Vu T.-A. (2011). Abdominal Migraine: An Under-Diagnosed Cause of Recurrent Abdominal Pain in Children. Headache J. Head Face Pain.

[28] Rothner A.D., Parikh S. (2016). Migraine Variants or Episodic Syndromes That May Be Associated with Migraine and Other Unusual Pediatric Headache Syndromes. Headache J. Head Face Pain.

[29] Lebron D., Vasconcellos E. (2016). The Episodic Syndromes That Maybe Associated with Migraines. Semin. Pediatr. Neurol..

[30] Spiri D., Rinaldi V.E., Titomanlio L. (2014). Pediatric migraine and episodic syndromes that may be associated with migraine. Ital. J. Pediatr..

[31] Prakash C., Staiano A., Rothbaum R.J., Clouse R.E. (2001). Similarities in cyclic vomiting syndrome across age groups. Am. J. Gastroenterol..

[32] Napthali K., Koloski N., Talley N.J. (2016). Abdominal migraine. Cephalalgia.

[33] Taché Y. (1999). Cyclic vomiting syndrome: The corticotropin-releasing-factor hypothesis. Dig. Dis. Sci..

[34] Dignan F., Abu-Arafeh I., Russell G. (2001). The prognosis of childhood abdominal migraine. Arch. Dis. Child..

[35] Catto-Smith A.G., Ranuh R. (2003). Abdominal migraine and cyclical vomiting. Semin. Pediatr. Surg..

[36] Redon S., Mareau C., Guedj E., Donnet A. (2017). Cyclic Vomiting Syndrome in Adults and Children: A Hypothesis. Headache J. Head Face Pain.

[37] Moavero R., Papetti L., Bernucci M.C., Cenci C., Ferilli M.A.N., Sforza G., Vigevano F., Valeriani M. (2019). Cyclic vomiting syndrome and benign paroxysmal torticollis are associated with a high risk of developing primary headache: A longitudinal study. Cephalalgia.

[38] Raucci U., Borrelli O., Di Nardo G., Tambucci R., Pavone P., Salvatore S., Baldassarre M.E., Cordelli D.M., Falsaperla R., Felici E. (2020). Cyclic Vomiting Syndrome in Children. Front. Neurol..

[39] Rashed H., Abell T.L., Familoni B., Cardoso S. (1999). Autonomic function in cyclic vomiting syndrome and classic migraine. Dig. Dis. Sci..

[40] Lin Y.-P., Ni Y.-H., Weng W.-C., Lee W.-T. (2011). Cyclic Vomiting Syndrome and Migraine in Children. J. Formos. Med Assoc..

[41] Boles R.G., Chun N., Senadheera D., Wong L.-J.C. (1997). Cyclic vomiting syndrome and mitochondrial DNA mutations. Lancet.

[42] Rinaldo P. (1999). Mitochondrial fatty acid oxidation disorders and cyclic vomiting syndrome. Dig. Dis. Sci..

[43] Chong S.K. (1999). Electrogastrography in cyclic vomiting syndrome. Dig. Dis. Sci..

[44] Hejazi R.A., Lavenbarg T.H., Pasnoor M., Dimachkie M., Foran P., Herbelin L., Mccallum R.W. (2011). Autonomic nerve function in adult patients with cyclic vomiting syndrome. Neurogastroenterol. Motil..

[45] Fajardo N.R., Cremonini F., Talley N.J. (2005). Frontiers in functional dyspepsia. Curr. Gastroenterol. Rep..

[46] Turchetti A., Guglielmi S., Fossati C., Matrunola M., Corrado G. (2005). Gastric emptying time in cyclic vomiting syndrome in children. Eur. Rev. Med. Pharmacol. Sci..

[47] Hejazi R.A., Lavenbarg T.H., Mccallum R.W. (2010). Spectrum of gastric emptying patterns in adult patients with cyclic vomiting syndrome: Gastric emptying in cyclic vomiting syndrome. Neurogastroenterol. Motil..

[48] Howlett A. (2002). International Union of Pharmacology. XXVII. Classification of Cannabinoid Receptors. Pharmacol. Rev..

[49] Pesce M., D’Alessandro A., Borrelli O., Gigli S., Seguella L., Cuomo R., Esposito G., Sarnelli G. (2017). Endocannabinoid-related compounds in gastrointestinal diseases. J. Cell. Mol. Med..

[50] Donnet A., Redon S. (2018). Cyclic Vomiting Syndrome in Children. Curr. Pain Headache Rep..

[51] Eidlitz-Markus T., Haimi-Cohen Y., Zeharia A. (2017). Vomiting and migraine-related clinical parameters in pediatric migraine. Headache J. Head Face Pain.

[52] Castro-Rodriguez J.A., Stern D.A., Halonen M., Wright A.L., Holberg C.J., Taussig L.M., Martinez F.D. (2001). Relation between infantile colic and asthma/atopy: A prospective study in an unselected population. Pediatrics.

[53] Lucassen P., Assendelft W., Van Eijk J.T.M., Gubbels J., Douwes A., Van Geldrop W. (2001). Systematic review of the occurrence of infantile colic in the community. Arch. Dis. Child..

[54] Gelfand A., Goadsby P.J., Allen I.E. (2015). The relationship between migraine and infant colic: A systematic review and meta-analysis. Cephalalgia.

[55] Jan M.M.S., Al-Buhairi A.R. (2001). Is Infantile Colic a Migraine-Related Phenomenon?. Clin. Pediatr..

[56] Lucassen P.L.B.J., Assendelft W.J.J., Gubbels J.W., Van Eijk J.T.M., Van Geldrop W.J., Neven A.K. (1998). Effectiveness of treatments for infantile colic: Systematic review. BMJ.

[57] Algranati P.S., Dworkin P.H. (1992). Infancy Problem Behaviors. Pediatr. Rev..

[58] Romanello S., Spiri D., Marcuzzi E., Zanin A., Boizeau P., Riviere S., Vizeneux A., Moretti R., Carbajal R., Mercier J.-C. (2013). Association Between Childhood Migraine and History of Infantile Colic. JAMA.

[59] Epstein L.G., Zee P.C. (2013). Infantile Colic and Migraine. JAMA.

[60] Lindskog U., Ödkvist L., Noaksson L., Wallquist J. (1999). Benign Paroxysmal Vertigo in Childhood: A Long-term Follow-up. Headache J. Head Face Pain.

[61] Batuecas-Caletrío Á., Martín-Sánchez V., Cordero-Civantos C., Guardado-Sánchez L., Marcos M.R., Fabián A.H., González J.J.B., Cruz-Ruiz S.S. (2013). Is Benign Paroxysmal Vertigo of Childhood a migraine precursor?. Eur. J. Paediatr. Neurol..

[62] Koehler B. (1980). Benign paroxysmal vertigo of childhood: A migraine equivalent. Eur. J. Nucl. Med. Mol. Imaging.

[63] Drigo P., Carli G., Laverda A.M. (2001). Benign paroxysmal vertigo of childhood. Brain Dev..

[64] Shin M., Douglass L.M., Milunsky J.M., Rosman N.P. (2016). The Genetics of Benign Paroxysmal Torticollis of Infancy: Is There an Association with Mutations in the CACNA1A Gene?. J. Child Neurol..

[65] Ophoff R.A., Terwindt G.M., Vergouwe M.N., Frants R.R., Ferrari M.D. (1997). Involvement of a Ca^2+^ Channel Gene in Familial Hemiplegic Migraine and Migraine with and Without Aura. Headache J. Head Face Pain.

[66] Stendel C., D’Adamo M.C., Wiessner M., Dusl M., Cenciarini M., Belia S., Nematian-Ardestani E., Bauer P., Senderek J., Klopstock T. (2020). Association of A Novel Splice Site Mutation in P/Q-Type Calcium Channels with Childhood Epilepsy and Late-Onset Slowly Progressive Non-Episodic Cerebellar Ataxia. Int. J. Mol. Sci..

[67] Zhuchenko O., Bailey J., Bonnen P., Ashizawa T., Stockton D.W., Amos C., Dobyns W., Subramony S., Zoghbi H., Lee C.C. (1997). Autosomal dominant cerebellar ataxia (SCA6) associated with small polyglutamine expansions in the α1A-voltage-dependent calcium channel. Nat. Genet..

[68] Costa C., Prontera P., Sarchielli P., Tonelli A., Bassi M.T., Cupini L.M., Caproni S., Siliquini S., Donti E., Calabresi P. (2013). A novel ATP1A2 gene mutation in familial hemiplegic migraine and epilepsy. Cephalalgia.

[69] Coppola G., Pastorino G.M.G., Vetri L., D’Onofrio F., Operto F.F. (2020). Familial Hemiplegic Migraine with an ATP1A4 Mutation: Clinical Spectrum and Carbamazepine Efficacy. Brain Sci..

[70] Castro M.-J., Stam A., Lemos C., De Vries B., Vanmolkot K., Barros J., Terwindt G., Frants R., Sequeiros J., Ferrari M.D. (2009). First Mutation in the Voltage-Gated Nav1.1 Subunit Gene SCN1A with Co-Occurring Familial Hemiplegic Migraine and Epilepsy. Cephalalgia.

[71] Dale R.C., Gardiner A., Antony J., Houlden H. (2012). Familial PRRT2 mutation with heterogeneous paroxysmal disorders including paroxysmal torticollis and hemiplegic migraine: Case Report. Dev. Med. Child Neurol..

[72] Murdin L., Chamberlain F., Cheema S., Arshad Q., Gresty M., Golding J.F., Bronstein A. (2014). Motion sickness in migraine and vestibular disorders. J. Neurol. Neurosurg. Psychiatry.

[73] Von Brevern M. (2004). Acute migrainous vertigo: Clinical and oculographic findings. Brain.

[74] Cutrer F.M., Baloh R.W. (1992). Migraine-associated Dizziness. Headache J. Head Face Pain.

[75] Brey R.L. (2005). Both migraine and motion sickness may be due to low brain levels of serotonin. Neurology.

[76] Kayan A., Hood J.D. (1984). Neuro-otological manifestations of migraine. Brain.

[77] Cuomo-Granston A., Drummond P.D. (2010). Migraine and motion sickness: What is the link?. Prog. Neurobiol..

[78] Mitchelson F. (1992). Pharmacological agents affecting emesis. A review (Part II). Drugs.

[79] Barabas G., Matthews W.S., Ferrari M. (1983). Childhood migraine and motion sickness. Pediatrics.

[80] Jan M. (1998). History of motion sickness is predictive of childhood migraine. J. Paediatr. Child Health.

[81] Gupta S.N., Gupta V.S., Borad N. (2015). Spectrum of migraine variants and beyond: The individual syndromes in children. Brain Dev..

[82] Guiloff R.J., Fruns M. (1988). Limb pain in migraine and cluster headache. J. Neurol. Neurosurg. Psychiatry.

[83] Guiloff R.J., Fruns M. (1990). Migrainous Limb Pain. A Historical Note. Headache J. Head Face Pain.

[84] Raudino F. (1994). Limb Pain and Headache. Headache J. Head Face Pain.

[85] Saito Y., Fusayasu E., Iitsuka T., Takeshima T., Ohno K. (2006). Familial limb pain in childhood: Unusual manifestation of migraine?. Brain Dev..

[86] Piovesan E.J., Young B.W., Werneck L.C., Kowacs P., Oshinsky M.L., Silberstein S.D. (2003). Recurrent Extratrigeminal Stabbing and Burning Sensation with Allodynia in A Migraine Patient. Cephalalgia.

[87] Cuadrado M., Young W., Fernández-De-Las-Peñas C., Arias J., Pareja J. (2008). Migrainous Corpalgia: Body Pain and Allodynia Associated with Migraine Attacks. Cephalalgia.

[88] Angus-Leppan H., Guiloff R.J. (2016). Familial limb pain and migraine: 8-year follow-up of four generations. Cephalalgia.

[89] Le Gal J., Michel J.-F., Rinaldi V.E., Spiri D., Moretti R., Bettati D., Romanello S., Berlese P., Lualdi R., Boizeau P. (2016). Association between functional gastrointestinal disorders and migraine in children and adolescents: A case-control study. Lancet Gastroenterol. Hepatol..

[90] Weydert J.A., Ball T.M., Davis M.F. (2003). Systematic Review of Treatments for Recurrent Abdominal Pain. Pediatr..

[91] Frattale I., Caponnetto V., Casalena A., Assetta M., Maddestra M., Marzoli F., Affaitati G., Giamberardino M.A., Viola S., Gabriele A. (2021). Association between response to triptans and response to erenumab: Real-life data. J. Headache Pain.

[92] Tiseo C., Ornello R., Pistoia F., Sacco S. (2019). How to integrate monoclonal antibodies targeting the calcitonin gene-related peptide or its receptor in daily clinical practice. J. Headache Pain.

[93] Sun J., Wu X., Meng Y., Cheng J., Ning H., Peng Y., Pei L., Zhang W. (2015). Electro-acupuncture decreases 5-HT, CGRP and increases NPY in the brain-gut axis in two rat models of Diarrhea-predominant irritable bowel syndrome(D-IBS). BMC Complement. Altern. Med..

[94] Sándor K., Kormos V., Botz B., Imreh A., Bölcskei K., Gaszner B., Markovics A., Szolcsányi J., Shintani N., Hashimoto H. (2010). Impaired nocifensive behaviours and mechanical hyperalgesia, but enhanced thermal allodynia in pituitary adenylate cyclase-activating polypeptide deficient mice. Neuropeptides.

[95] Kim D.-Y., Camilleri M. (2000). Serotonin: A Mediator of The Brain–Gut Connection. Am. J. Gastroenterol..

[96] Gormley P., Anttila V., Winsvold B.S., Palta P., Esko T., Pers T.H., Farh K.-H., Cuenca-Leon E., Muona M., International Headache Genetics Consortium (2016). Meta-analysis of 375,000 individuals identifies 38 susceptibility loci for migraine. Nat. Genet..

[97] Strassman A.M., Raymond S.A., Burstein R. (1996). Sensitization of meningeal sensory neurons and the origin of headaches. Nat. Cell Biol..

[98] Bernstein C., Burstein R. (2012). Sensitization of the Trigeminovascular Pathway: Perspective and Implications to Migraine Pathophysiology. J. Clin. Neurol..

[99] Goadsby P., Holland P., Martins-Oliveira M., Hoffmann J., Schankin C., Akerman S. (2017). Pathophysiology of Migraine: A Disorder of Sensory Processing. Physiol. Rev..

[100] Badihian N., Saneian H., Badihian S., Yaghini O. (2018). Prophylactic Therapy of Cyclic Vomiting Syndrome in Children: Comparison of Amitriptyline and Cyproheptadine: A Randomized Clinical Trial. Am. J. Gastroenterol..

[101] Lee L., Abbott L., Mahlangu B., Moodie S. (2012). PWE-054 The management of cyclic vomiting syndrome: A systematic review of 1141 cases: Abstract PWE-054 Table 1. Gut.

[102] Kakisaka Y., Wakusawa K., Haginoya K., Saito A., Uematsu M., Yokoyama H., Sato T., Tsuchiya S. (2009). Efficacy of Sumatriptan in Two Pediatric Cases with Abdominal Pain-Related Functional Gastrointestinal Disorders: Does the Mechanism Overlap That of Migraine?. J. Child Neurol..

[103] Kothare S.V. (2005). Efficacy of flunarizine in the prophylaxis of cyclical vomiting syndrome and abdominal migraine. Eur. J. Paediatr. Neurol..

[104] Valeriani M., Galli F., Tarantino S., Graceffa D., Pignata E., Miliucci R., Biondi G., Tozzi A.E., Vigevano F., Guidetti V. (2009). Correlation Between Abnormal Brain Excitability and Emotional Symptomatology in Paediatric Migraine. Cephalalgia.

[105] De Tommaso M., Ambrosini A., Brighina F., Coppola G., Perrotta A., Pierelli F., Sandrini G., Valeriani M., Marinazzo D., Stramaglia S. (2014). Altered processing of sensory stimuli in patients with migraine. Nat. Rev. Neurol..

[106] Evers S., Bauer B., Grotemeyer K.-H., Kurlemann G., Husstedt I.-W. (1998). Event-related potentials (P300) in primary headache in childhood and adolescence. J. Child Neurol..

[107] Buodo G., Palomba D., Sarlo M., Naccarella C., Battistella P. (2004). Auditory Event-Related Potentials and Reaction Times in Migraine Children. Cephalalgia.

[108] Zohsel K., Hohmeister J., Flor H., Hermann C. (2008). Altered pain processing in children with migraine: An evoked potential study. Eur. J. Pain.

[109] Kropp P., Siniatchkin M., Stephani U., Gerber W.-D. (1999). Migraine—Evidence for a disturbance of cerebral maturation in man?. Neurosci. Lett..

[110] Oelkers-Ax R., Parzer P., Resch F., Weisbrod M. (2005). Maturation of Early Visual Processing Investigated by a Pattern-Reversal Habituation Paradigm is Altered in Migraine. Cephalalgia.

[111] Koute V., Michalopoulou A., Siokas V., Aloizou A.-M., Rikos D., Bogdanos D.P., Kontopoulos E., Grivea I.N., Syrogiannopoulos G.A., Papadimitriou A. (2021). Val66Met polymorphism is associated with decreased likelihood for pediatric headache and migraine. Neurol. Res..

[112] Nijs J., Meeus M., Versijpt J., Moens M., Bos I., Knaepen K., Meeusen R. (2014). Brain-derived neurotrophic factor as a driving force behind neuroplasticity in neuropathic and central sensitization pain: A new therapeutic target?. Expert Opin. Ther. Targets.

[113] Obata K., Noguchi K. (2006). BDNF in sensory neurons and chronic pain. Neurosci. Res..

[114] Ichikawa H., Yabuuchi T., Jin H., Terayama R., Yamaai T., Deguchi T., Kamioka H., Takano-Yamamoto T., Sugimoto T. (2006). Brain-derived neurotrophic factor-immunoreactive primary sensory neurons in the rat trigeminal ganglion and trigeminal sensory nuclei. Brain Res..

[115] Guarnera A., Bottino F., Napolitano A., Sforza G., Cappa M., Chioma L., Pasquini L., Rossi-Espagnet M.C., Lucignani G., Figà-Talamanca L. (2021). Early alterations of cortical thickness and gyrification in migraine without aura: A retrospective MRI study in pediatric patients. J. Headache Pain.

[116] Abu-Arafeh I., Gelfand A.A. (2021). The childhood migraine syndrome. Nat. Rev. Neurol..

